# Supplementary material for: Are carbon emissions trading and green financial instruments synergistic? -Comprehensive quantitative research based on content analysis
Source: PLoS One. 2024 Mar 7;19(3):e0298601. doi: 10.1371/journal.pone.0298601 (PMC10919601; doi:10.1371/journal.pone.0298601)
Supplement: S1 Data — (ZIP) [file pone.0298601.s001.zip › Data/地级市Z_F工作报告.zip!/╕ú╜¿/─╧╞╜╩╨/└┤╫╘╣1⁄2╓┌║┼í╛┬φ┐╦╩2╛▌═°í┐_2019.docx]

各位代表：

现在，我代表南平市人民政府，向大会报告政府工作，请予审议，并请市政协各位委员和其他列席人员提出意见。

一、2018年主要工作回顾

2018年是全面贯彻落实党的十九大精神开局之年，是改革开放40周年。在以习近平同志为核心的党中央坚强领导下，我市各级政府坚持以习近平新时代中国特色社会主义思想为指引，牢记习近平总书记对南平的殷切嘱托，认真贯彻党的十九大和十九届二中、三中全会精神，全面落实省委十届六次、七次全会和市委五届六次全会作出的决策部署，紧紧围绕加快绿色发展推动高质量发展落实赶超这一战略目标，坚持新发展理念，坚持以供给侧结构性改革为主线，落实“六稳”工作要求，有效应对外部环境深刻变化，深化“四比六促”“项目突破年”活动，加快实施“八项行动”，坚决打好三大攻坚战，经济保持平稳发展，社会事业全面进步，人民群众安居乐业。

初步统计，2018年全市生产总值1792.51亿元、增长6.6%；固定资产投资增长10.1%；一般公共预算总收入144.14亿元、增长11%；地方一般公共预算收入94.52亿元、增长8.5%；外贸出口98.3亿元、增长16.6%，实际利用外资10.3亿元；社会消费品零售总额675.09亿元、增长9.7%；城镇居民、农村居民人均可支配收入32484元、15868元，分别增长8%、9%。全市单位GDP能耗、空气质量优良天数比例、主要流域水质优良比例和4项主要污染物总量控制指标完成省委、省政府下达的任务。一年来的主要工作和成效是：

（一）项目突破卓有成效。坚持把项目建设作为经济增长的重要抓手，实行“市领导挂点重点项目”“千名干部服务千个重点项目”机制，全市新增“五个一批”项目2785项，总投资5587亿元。南平和顺昌、邵武分别获得全省“五个一批”项目正向激励综合考评奖励。强力推进项目集中开竣工，46个在建赶超重大项目完成投资105.35亿元，355个在建重点项目完成投资604.28亿元，37个投资工程包完成投资138亿元，巨电新能源、元力高端精制活性炭、永晶新材料等一批重点产业项目建成投产，南龙铁路南平段、武夷新区绕城高速和303线快速通道等一批重大交通基础设施建成通车，顺邵高速将于1月中旬通车，全市高速公路通车里程突破1000公里。大力推进“一把手”招商，积极参加首届进博会、首届数字中国建设峰会、“6·18”项目成果交易会、“9·8”投洽会、2018两岸企业家峰会，成功举办第14届中国茶业经济年会、第12届海峡两岸茶博会、第2届中国（武夷）生态食品博览会，引进建阳竹缠绕复合管道、邵武新宙邦高性能氟材料、浦城蒙正生物制药、光泽现代渔业产业园等总投资超10亿元的项目27个，与人工智能领军企业商汤集团签订战略合作协议，发展后劲和动能进一步增强。

（二）绿色产业提质增效。全面加快落实绿色产业发展行动纲要和七大产业规划，42个先行工程和294个支撑项目加快推进，七大产业完成投资占固投的56%；七大产业规上企业增至880家，占全市规上企业的79.2%，竞争力进一步提升。坚持创新驱动，研发投入占GDP比重提高到1.03%，新认定国家知识产权优势企业5家、科技小巨人领军企业19家。全市高新企业发展至47家，科技型中小企业100家，高技术产业增加值增长27.79%。实施品牌战略，成功举办武夷品牌建设发布会，制定武夷品牌战略规划，打造“武夷山水”区域公用品牌，武夷山矿泉水、明良食品等8家企业获得授权使用。新认定驰名商标1件、地理标志证明商标21件。圣农成为亚洲最大的白羽肉鸡产销基地，上榜中国民营企业500强。突出实体经济，对接省“百千万”行动，召开全市工业发展大会，弘扬企业家精神，出台进一步促进企业发展、加快培育重点龙头企业和行业小巨人领军企业促进产业集群发展等扶持措施，持续开展“百名局长帮扶百家企业”活动，一企一策精准帮助企业降低成本、开拓市场、上市融资，积极对接引进上下游企业，延伸产业链，着力解决一批工业企业历史遗留问题。做优第三产业，大力培育“互联网+”新业态、新模式，商贸物流、电子商务等服务业加快发展。旅游产业转型提升，武夷山五夫朱子文化园、顺昌宝山旅游风景区等一批旅游项目加快推进，新增省级旅游休闲基地3个、3A级以上旅游景区10家，旅游产品供给不断丰富。全市旅游接待总人数4980万人次、增长20%，旅游总收入770亿元、增长30%。

（三）“三大攻坚战”取得阶段性成果。始终坚持把“三大攻坚战”摆在全年工作的重要位置，坚持问题导向，聚焦重点，综合施策。防范化解重大风险措施有力。强化政府债务限额管理，严厉打击和处置非法集资、逃废债、虚假租赁等违法行为，全市化解不良贷款31.52亿元，不良贷款率降至2.07%。精准脱贫扎实有效。深化挂钩帮扶制度，现有建档立卡贫困户实现体制内干部结对帮扶全覆盖，346个贫困村实现驻村第一书记全覆盖。实施产业扶贫“五个一百”示范带动工程和“百企帮百村”活动，落实精准医疗扶贫慈善救助制度，实施“雨露计划”“春潮行动”，1.15万贫困劳动力实现就业。造福工程易地扶贫搬迁1836人，超额完成省下达任务。5个省级扶贫开发工作重点县1个“脱帽”、3个申请“脱帽”，207个建档立卡贫困村脱贫出列，2017年底新增贫困和返贫人口达到脱贫退出标准。生态环境质量保持在全省前列。坚持最严的生态标准，继续实施“清新水域”“洁净蓝天”“清洁土壤”工程，厚植绿色生态优势。坚决落实河湖长制，畜禽养殖污染专项整治成果继续巩固，3条主要河流Ⅰ—Ⅲ类水质比例100%，15个集中式生活饮用水水源地水质达标率100%，区域内小流域劣V类水体全面消除；坚持臭氧和PM2.5协同控制，严控高耗能高排放项目建设，空气优良天数比例达99%；加大水土流失综合治理力度，完成水土流失治理40.69 万亩，整治毁林种茶2.8万亩。落实“三个必造”，复绿造林1.34万亩，森林覆盖率提升至77.99%。扎实推进国家生态文明试验区建设，武夷山国家公园体制试点区自然资源统一确权登记工作通过验收，闽江流域山水林田湖草生态保护修复、国家储备林质量精准提升、武夷山田园综合体试点工程稳步实施。

（四）“水美城市”加快推进。坚持“水安为先，因水而美、由美而富、富而文明”的理念，持续加快建设“水美城市”，完成投资62.54亿元，各县（市、区）建成一批滨水公园、栈道等项目，城市面貌焕然一新，城市品质和品位显著提升。南平市和3个县（市、区）获得省级文明城市（县城、城区）称号，6个县（市、区）获得省级森林城市称号。启动行政中心搬迁，武夷新区加快建设各类基础设施和公共服务设施，核心区综合路网等一批基础设施投入使用，“天圆地方”、政务服务中心等公共服务设施主体工程建设基本完成，万达综合体正式开业运营，云谷小区、体育中心等重点项目加快实施，南平一中武夷新区高中部、少年儿童重点业余体育学校等项目开工建设，公交系统逐步向南林核心区延伸，武夷新区城市格局初步形成。建阳区加快实施嘉禾大道拓宽改造、双龙桥隧等57项、总投资103.3亿元的同城化项目，与武夷新区紧密相融。延平城区建设同步推进，“三江六岸”景观提升工程加快实施，康体休闲慢行段、滨水花海景观段、古渡公园段对市民开放，九峰山空中栈道、九峰隧道、杨真隧道加快建设，继续实施一批立面改造、道路“白改黑”和地下污水管网改造提升项目，夜景亮化工程投入使用。闽江航道整治、新港路二期、316国道改扩建及瓦口洋湿地公园等项目加快推进，城市公共服务设施和功能配套不断完善。

（五）乡村振兴战略稳步实施。高标准编制《南平市乡村振兴战略规划》，全面实施“十大工程”，着力推动“五大振兴”。 加快推进国家农业可持续发展试验示范区建设，新发展农民专业合作社837个、家庭农场653家，新增高标准农田28.26万亩，建成3个国际标准农产品示范基地，完成市本级农产品检测中心建设，对重要农产品推行“一品一码”销售。深化“南平机制”，创新科技特派员选派、联动、服务、保障机制，在派科技特派员1672人，连续19年推进科技特派员工作被国务院第五次大督查通报表扬。深化农村改革，农垦国有土地确权登记发证17万亩，1652个村开展集体产权制度改革。大力实施农村人居环境整治三年行动，推进“一革命四行动”，新改建农村公厕257座，新建改造村庄三格化粪池3.6万户，119个省级美丽乡村新增绿化面积7.7万平方米、整治裸房2130栋，建成精品示范村25个。落实路长制，加快建设“四好农村路”，新改建农村公路485公里。开展移风易俗行动，树立文明乡风。严格标准、严明纪律，村级组织换届选举顺利完成。

（六）改革开放不断深化。改革开放40年来，南平始终坚持解放思想、实事求是，坚持以经济建设为中心，不断解放和发展社会生产力，闽北大地发生了翻天覆地变化，GDP增长202倍，公共财政收入增长112倍, 社会消费品零售总额增长了202倍。40年的实践充分证明，唯有改革开放才能加快发展。2018年，南平坚持把改革开放作为加快绿色发展的强大动力，继续高举改革开放伟大旗帜。深入推进“放管服”改革，“五级一体”的“互联网+放管服”云平台和网上公共服务平台不断完善，在全省率先实现“双随机一公开”监管全覆盖，全市“一趟不用跑”和“最多跑一趟”审批服务事项占总事项数的93%以上，4个政务服务标准获省级地方标准立项审定。商事制度改革取得新突破，企业开办时间压缩到5个工作日内，新增市场主体4.48万户。国有资产管理体制日趋完善，实现清单式监管。财税改革进一步推进，预决算公开规范化、常态化、制度化，预算绩效管理扩面提质。健全国家自然资源资产管理体制试点工作有序推进，摸清了自然资源家底，实现信息化管理。积极推进“生态银行”建设，组建生态资源保护开发有限公司和专家委员会，顺昌成立首家“森林生态银行”，武夷山五夫镇成立生态资源营业部，策划实施了顺昌西坑林业综合体、延平巨口艺术旅游等项目。完成监察体制改革，有序推进市县机构改革、事业单位分类改革。积极融入“一带一路”发展战略，主动加强闽东北协同发展区、闽浙赣皖福州经济协作区等经贸交流合作，12个总投资551.5亿元的省区域协作重大项目加快实施。推动口岸扩大开放，武夷山航空口岸临时开放获国家口岸办批准，武夷山—曼谷首条国际航线正式通航，武夷山陆地港获批设立中欧班列装卸站点，对外开放水平进一步提升。

（七）社会民生持续改善。民生支出占地方一般公共预算支出比重达83.49%，26项为民办实事项目全面完成。城镇新增就业2万人，城镇登记失业率2.87%，城乡低保标准一体化全面实现。教育五大提升工程加快实施，南平中学岭道路等一批教育配套设施投入使用，建成8所公办幼儿园，创建40所省级义务教育教改示范校、1所省级示范性高中，2018年高考20人进入全省前300名，本一上线人数3500多人，教育翻身仗初见成效。卫生服务不断优化，全面推进县域紧密型医共体建设，10个县（市、区）总医院均挂牌成立，80.2万人签订社区家庭医生服务协议。医疗保障进一步完善，实现城镇职工、城镇居民、新农合“三保合一”市级统筹。养老服务显著提升，城乡居民养老保险参保131万人，建成居家养老服务照料中心23个、农村幸福院203个。文体事业蓬勃发展，获得第十七届省运会承办权，成功举办纪念朱子诞辰888周年系列活动、武夷山国际马拉松赛、中国龙舟公开赛、闽浙赣皖四省四市民间艺术节等活动，文艺“五个一百”工程取得阶段性成效。社会更加安定和谐，广泛深入开展扫黑除恶专项斗争和社会治安“五个专项治理”，扎实推进“七五”普法，加强信访维稳工作，强化安全生产和食品药品监管，群众安全感明显提升。妇女儿童发展纲要全面实施，民族宗教、外事侨务、台港澳事务、气象防震、人民防空、档案方志、老区库区等工作继续加强，群团改革深入推进。全力支持国防和军队建设，军政军民关系融洽和谐。

（八）政府自身建设进一步加强。始终坚持以党的政治建设为统领，牢固树立“四个意识”，坚定“四个自信”，落实“两个维护”，持续兴起习近平新时代中国特色社会主义思想“大学习”热潮，扎实开展向廖俊波同志学习活动，有力推进“两学一做”学习教育常态化制度化。全面落实从严治党的主体责任和监督责任，把落实中央巡视整改反馈意见作为重大政治任务，主动认领问题，细化整改措施，各项整改工作取得明显成效。按照省委“八个坚定不移”的要求，坚持在市委的领导下开展工作，严格落实“三重一大”决策制度，定期向市委常委会汇报工作。法治政府建设不断推进，组织起草并提请市人大审议通过《南平市饮用水水源保护办法》《南平市河岸生态地保护规定》和《南平市城市绿地管理办法》等法规。自觉接受人大监督、政协监督、监察监督和社会监督，办理人大代表建议议案218件，政协委员提案291件，满意率分别为99.23%、99.65%；创新建立“随手拍”社会化监督机制，实现“零距离”全民监督。作风建设持续深化，严格落实中央八项规定及其实施细则精神和省市实施办法，进一步弘扬“马上就办、真抓实干”“四下基层”优良作风；电子政务综合试点稳步推进，行政效能、机关效能进一步提升，“两单”平台被列为全国政府系统督查信息化建设优秀典型之一。加强审计监督，全面推进政务公开和权力运行网上公开，党风廉政建设和反腐败工作向纵深发展。

各位代表，过去一年，路途充满艰辛，成绩来之不易。这是习近平新时代中国特色社会主义思想指引的结果，是省委、省政府和市委总揽全局、正确领导的结果，是市人大、市政协有效监督、大力支持的结果，是全市人民齐心协力、团结拼搏的结果。在此，我代表市人民政府，向全市人民，向各民主党派、工商联、各人民团体和各界人士，向中央和国家机关以及省直机关驻地机构，向人民解放军和武警驻地部队，向关心支持南平发展的台港澳同胞、海外乡亲和国际友人，致以崇高的敬意和衷心的感谢！

成绩令人鼓舞，问题不容忽视。前进的道路上，我们还面临诸多困难挑战，发展中还存在着不少问题：产业规模不大，农业种养业比重高、比较效益低、市场竞争力不强，战略性新兴产业刚刚起步，现代服务业有待提质壮大；企业“小弱散”问题没有得到有效解决，缺乏带动力强的行业龙头企业和大项目；自主创新能力不强，高新技术企业、新型研发机构、科技企业孵化器与发达地区相比差距较大，人才总量特别是高层次人才还不能满足高质量发展的需要；公共服务与群众期待还有不少差距，保障和改善民生仍需做大量工作；城市规划建设管理水平有待提升；部分干部适应新时代新经济发展的能力不强，拼闯的冲劲和担当的精神不足。对于这些困难和问题，我们一定勇于直面、迎难而上，采取有力措施，切实加以解决，努力把各项工作做得更好更扎实。

二、奋起直追、落实赶超，全力以赴做好2019年工作

今年是新中国成立70周年，是决胜全面建成小康社会、坚持绿色发展推动高质量发展的关键一年，落实赶超任务尤为重要、尤为紧迫。综合分析国内外形势，发展面临的机遇和挑战并存，经济运行稳中有变、变中有忧，外部环境复杂严峻，经济面临下行压力。但我们要看到，南平有生态、区位等一系列优势，在新一轮经济发展浪潮中，我们一定要有不甘落后的志气、勇往直前的魄力，以更大的信心和决心、更多的智慧和创造、更足的干劲和勇气，主动应对宏观经济形势，盯紧目标、把握机遇，持续发力、久久为功，以钉钉子精神做实做细做好各项工作，努力实现高质量发展落实赶超。

今年政府工作的总体要求是：以习近平新时代中国特色社会主义思想为指导，全面贯彻党的十九大和十九届二中、三中全会精神，紧紧围绕统筹推进“五位一体”总体布局和协调推进“四个全面”战略布局，坚持稳中求进工作总基调，坚持新发展理念，坚持绿色发展推动高质量发展落实赶超，坚持以供给侧结构性改革为主线，坚持深化市场化改革、扩大高水平开放，继续打好三大攻坚战，加快实体经济创新转型，加快建设现代化经济体系，统筹推进稳增长、促改革、调结构、惠民生、防风险、保稳定工作，保持经济运行在合理区间，进一步稳就业、稳金融、稳外贸、稳外资、稳投资、稳预期，提振市场信心，增强人民群众获得感、幸福感、安全感，推动绿水青山转化为金山银山，加快建设全国绿色发展示范区，以优异成绩庆祝新中国成立70周年。

2019年经济社会发展的主要预期目标是：全市生产总值增长8%—8.2%，固定资产投资增长10%，一般公共预算总收入增长5%，地方一般公共预算收入增长4.5%，外贸出口增长3%，实际利用外资增长3%，社会消费品零售总额增长10%，城镇居民人均可支配收入增长8%，农村居民人均可支配收入增长8.6%。全市单位GDP能耗、空气质量优良天数比例、主要流域水质优良比例和4项主要污染物总量控制指标完成省委、省政府下达任务。围绕上述目标，我们要奋力抓好以下九个方面的工作：

（一）勠力同心、铆足干劲，做大做强实体经济

始终把发展经济的着力点放在实体经济上，组建七大绿色产业发展研究小组，逐个产业进行梳理、研究、谋划，把产业的现状分析透、产业的链条搞清楚，分行业编制发展路线图，进一步明确产业的发展方向、发展目标、发展措施，实行“一业一策”精准扶持，打造一批市场占有率高、经济效益好、带动力强的小巨人领军企业、重点龙头企业和产业集群。

着力推动制造业高质量发展。主动对接省千亿产业集群推进计划，瞄准产业链、价值链高端，做好建链、补链、延链、强链的文章，打造氟新材料、食品加工、专用汽车配件等一批产值超百亿的产业集群。加快实施小巨人领军企业培育计划，通过走“专精特新”的发展道路，提升核心竞争力，培育一批独角兽、单项冠军、隐形冠军企业。发挥龙头企业带动作用，鼓励现有骨干企业通过收购、兼并、联合等方式，引领带动上下游配套企业发展。支持龙泰家居、闽瑞环保、利树集团等企业上市融资，积极对接科创板。大力实施“双高”培育工程，建设先进制造与工业设计中心，加快推进100项总投资340亿元的智能制造、“两化”融合和工业投资技改项目，力争全市高新技术企业、科技型企业分别达到50家、120家。

着力打造工业园区。把发展专业园区作为吸引投资和承接产业的主阵地，建立科学合理的利益分配机制，引导各县（市、区）共同参与各专业园区建设，促进园区做强做大。进一步明确园区产业发展定位，按照“土地集约、企业集聚、可持续发展”的原则和“大项目-产业链-产业群-产业基地”的路径，建设相应的产业服务配套设施，加强园区教育医疗、商业服务等综合功能培育，提升园区的承载力和竞争力。将土地运营、园区物业、物流管理与产业开发结合起来，进一步清理整顿闲置土地，处置“僵尸企业”，积极盘活低效利用的存量土地，提高土地利用率，提升园区投入产出比。

着力优化营商环境。更加关注民营企业、本土企业发展，坚持“两个毫不动摇”“三个没有变”，弘扬企业家精神、工匠精神，提供更加精准的政策供给，持续开展“百名局长帮扶百家企业”等活动，建立“政企直通车”机制，营造“亲”“清”新型政商关系。优化金融供给，开展“一对一”金融服务对接，落实无还本续贷、循环贷款等政策，推广“供应链融资”等模式，积极争取省级纾困基金和专项债，为企业提供多元化金融服务。发挥异地商会作用，成立专门的工作机构，大力实施“回归工程”，推动人才回归、资金回流、企业回迁。强化人力资源保障，实施更加开放的引才引智政策，鼓励大学生和外出务工、经商人员回乡就业创业。

（二）注重效益、持续发力，扩大精准有效投资

深入开展“项目提升年”活动，深化“五个一批”项目工作机制，扎实抓好447个省市重点项目，完成投资478亿元以上。

优化投资结构。坚持“补短板、调结构、促创新、惠民生”，把有限的资金投到能够增加有效供给、补齐发展短板的领域，投到具有引领性、高成长性的大项目上，投到能够促进发展的产业平台和基础设施配套上。认真谋划一批技术领先的先进制造业、战略性新兴产业和现代服务业项目，以及一批打基础、管长远的重大基础设施项目。全力推进温武吉铁路、昌福（厦）铁路、宁德至南平铁路、上浦高速、浦松高速、武夷山机场迁建等一批重大项目前期工作，加快建设衢宁铁路、武沙高速、邵武永和新型环保制冷剂、武夷山茶文化展销中心等重大项目，不断夯实发展基础。进一步激发民间投资活力，支持民间投资创新发展，鼓励民间资本投向“中国制造 2025”、七大绿色产业等领域。

细化项目管理。加强项目与重大政策、规划的衔接，科学编制有效投资三年滚动计划，合理安排重点建设项目，确保项目接续有力。对列入国家重大建设项目库、“五个一批”项目库的项目数据进行分析运用，持续开展项目集中开竣工活动，加强对已开工项目的考核管理，确保项目真正落地建设投产。

强化要素保障。完善项目协调推进机制，建立问题跟踪管理台账，及时协调解决项目推进过程中存在的困难和问题。进一步精简审批、核准等事项，加快投资项目审批承诺制改革。强化用地、用林、资金等要素综合保障，营造良好的项目成长环境。

（三）立足特色、发挥优势，推动服务业提档升级

以开拓创新的思路引领服务业发展，加快构筑与城市功能相适应、与区域经济相配套的产业体系。

大力发展现代服务业。突出发展文化创意产业，依托朱子文化、闽越文化、红色文化、茶文化等特色文化资源，加快五夫朱子文化园、考亭大宋文化生态书苑、建盏文化创意产业园等项目建设，培育壮大建盏、建本、点茶、根艺等产业。加快发展健康养生产业，规划建设一批健康产业园区，创新融医疗、康复、预防于一体的全链条服务模式，建设健康养生大数据平台，加快推进德懋堂、荣昌汇等项目，着力构建具有国际先进水平的特色健康养生产业高地。大力发展物流产业，加快建设武夷新区物流园、荣华山物流园以及南平市快递分拨中心、公共物流场站和信息平台，打造区域商品集散地。促进商贸业发展，统筹城乡商业网点的功能和布局，打造一批商圈及商贸综合体、专业商贸街区，改造和建设一批农产品批发市场和城乡集贸市场。鼓励发展家政服务连锁经营，整合家政服务社会资源，着力培育特色家庭服务品牌。提升金融服务水平，支持现有金融机构合理增加分支机构与营业网点，大力引进境内外金融机构在我市设点展业。

持续做优大武夷旅游。创新旅游体制机制，组建旅游产业发展基金，探索将国有旅游景区所有权、经营权分离，做活做大做强武夷山旅游龙头。加大市场营销力度，针对高铁城市圈、主要航线通达城市开展精准营销，办好第三届旅发大会，借助OTA平台提升大武夷旅游影响力。丰富产品供给，加快建设佳龙文旅、香江茶人小镇创客中心等一批差异化旅游项目，支持和平古镇争创5A级旅游景区，持续推出一批文旅、茶旅结合等体验类旅游产品，进一步推动武夷山由观光型旅游向休闲度假型旅游目的地转变。加快完善旅游基础设施，开发智慧旅游监管和大数据展示平台，推进旅游公路建设，新改建64座旅游厕所，建设光泽、浦城旅游集散服务中心，提升旅游通达性和便捷性。

（四）把握方向、重点突破，培育赶超发展新动能

以更大的魄力和决心，打造高端化、智能化、绿色化的发展新支撑，切实推动高质量发展。

以招商引资增强发展后劲。明确招商方向，梳理现有产业链条，围绕城市定位和产业规划开展精准招商，紧盯长三角、珠三角和闽南沿海地区，积极承接发达地区的外溢项目，多抱一些“金娃娃”。深化“一把手”招商，主动融入福南宁区域招商，创新招商方式，研究出台配套招商政策，深入开展基金招商、以商招商、委托招商、以投代招，积极参加“5·18”“6·18”“9·8”等各类经贸活动，办好茶博会、食博会，建立重大招商项目推进机制，力争年内新引进总投资超5000万元合同项目350个。

以新经济新产业释放发展活力。立足生态优势，牢牢把握新经济新产业发展的新机遇，积极研究其发展规律，建立新兴产业培育推进机制，实施新兴产业倍增计划，成立新兴产业孵化基金，加快推动新一代电子信息、人工智能、集成电路、大数据、云计算、新材料、新能源、节能环保、生物等产业集群化发展，力争战略性新兴产业增加值达110亿元。大力发展数字信息产业，依托武夷智谷软件园，加快推动商汤集团、恩蕊科技、硅谷数模、中电光谷等一批高新科技产业项目实施，着力打造云计算中心、大数据双创中心等应用孵化平台，推动数字产业化、产业数字化。突出在制造、金融、医疗、安防、政务等领域，下大力气抓一批人工智能重点应用示范项目和深度应用场景，推动人工智能与经济社会发展深度融合。积极发挥中国空间技术研究院南平军民融合产业发展中心的作用，大力实施卫星应用、特种车等一批军民融合重大产业项目，推动军民融合产业规模化发展。

以“生态银行”激发内生动力。发挥专家委员会战略指导作用，推动自然资源全域化整合、多元化增值，完善“生态银行”为实体企业提供原材料供应、担保增信、资产出租、市场准入认证等服务，积极发展单株林业碳汇交易等收益模式，不断拓展“生态银行”与产业运营方的合作方式。优化“生态银行”公司的股权结构，引入专业管理团队，提升市场化程度。加快与国内外知名产权股权交易机构、金融机构、产业投资运营商等建立广泛的合作关系，组建“生态银行”产业基金，增强产业发展导引能力。

以武夷品牌提升产业竞争力。全力落实武夷品牌发展战略规划，明晰“武夷山水”公共品牌定位，加快健全品控体系，积极将“武夷山水”打造成为全国生态精品农产品区域公用品牌，并推动其由农业向其他产业延伸。支持企业实施名牌战略、质量提升工程和标准化建设，全年新增中国驰名商标1件、注册地理标志证明商标10件。

（五）优化格局、提升标准，加快建设“水美南平”

拓展“水美城市”的内涵和外延，推动“水美城市”向“水美南平”延伸，进而发展“水美经济”。

全面展示城市形象。统筹流域间县（市、区）的规划衔接互补，加快编制《“水美南平”现代化规划》，打造山水相依、城景相融、人文厚重的宜居环境。用市场化理念发展“水美经济”，全年投资80亿元，新建或提升一批城市滨江景观带，新改建城市道路120公里、绿道130公里、管网480公里，进一步完善城市功能，改善城市自然景观风貌。以争创全国文明城市为目标，推进城市管理网格化、智慧化、法治化。注重工程质量，坚持精益求精，打造精品工程、百年工程，展示南平的水美、城美、人文美、生态美。

全速建设武夷新区。将武夷新区作为经济社会发展新引擎，进一步理顺市区管理、财税、运营和行政执法体制，加快行政中心搬迁。按照建设绿色城市、智慧城市、人文城市的要求，围绕南拓北联，拉开城市框架，积极推进武夷新区与建阳同城化发展、与武夷山度假区协同发展。推进金融CBD、南林核心区崇阳溪两岸等重要节点城市设计，加快云谷小区二期、商务写字楼等项目建设，逐步完善核心区功能要素。统筹布局公建设施，持续加快建设“十纵十横”交通路网、公交总站、体育中心、学校、医院以及园区生活配套等项目，做到与建阳区共建共享；着重在兴田片区实施一批会议会展、文化旅游、度假养生等产业项目和市政基础设施项目，加快与武夷山互利互融。坚持把城市规划定位与产业发展方向有机结合，持续做大浪潮大数据、巨电新能源、海源碳纤维车身、闽铝轻量化等项目，着力引进动力电芯、整车制造等项目，加快正新储能车等项目落地，实现产业链延伸，为城市发展提供强有力的支撑。

持续建设延平城区。坚持老城补短板，继续实施老旧小区整治提升和地下老旧管网治理改造工程，建设一批停车场、农超市场、城市公园等项目，加快实施主城区东进西扩规划，进一步完善公共服务设施。坚持新城强功能，完善学校、医院、公园等配套，结合316国道改扩建、南福路快速通道建设，打造商贸走廊；推进南平工业园区陈坑瓦口工业平台开发，完善生产生活配套，促进产城融合。加快建设南平港延平新城港区、九峰和杨真隧道、“三江六岸”景观提升、瓦口洋湿地公园、延平湖旅游综合开发、马头山—九峰山公园等项目，促进新老城区互联互通互动。

（六）真抓实干、强基固本，实施乡村振兴战略

按照“产业兴旺、生态宜居、乡风文明、治理有效、生活富裕”的总要求，实施乡村振兴五年建设规划，推动农业全面升级、农村全面进步、农民全面发展。

坚决打赢脱贫攻坚战。把脱贫攻坚作为实施乡村振兴战略的优先任务，深化挂钩帮扶，以一鼓作气、攻城拔寨的决心，全面完成脱贫攻坚任务，2018年新增贫困户全部脱贫，全市所有建档立卡贫困村实现脱贫出列，政和县实现“脱帽”。坚持“输血”与“造血”并重，大力实施“六大工程”，建立创业支持服务机制，搭建金融、电商、就业等服务平台，增强贫困户增收脱贫的内生动力。持续完善社会扶贫机制，充分发挥各类市场主体、社会组织和社会各界力量的作用，形成扶贫开发工作合力。

加快发展特色现代农业。深化农业供给侧结构性改革，持续推进国家农业可持续发展试验示范区建设，做大做强茶叶、果蔬、笋竹、食用菌、种业、畜禽、水产等特色农业。全面落实粮食安全责任制，稳步提升粮食综合生产能力。实施特色现代农业“五百工程”，农业产业化龙头企业、优质农产品标准化示范基地、农产品初加工中心、“三品一标”农产品和“一村一品”特色产业示范村分别新增20个以上。推广“五节一循环”模式，加强优质绿色、生态友好、节本增效品种技术研发推广，构建农业绿色生态产业链。推进农产品质量安全全程控制和监管能力建设，逐步建立农产品质量安全档案及追溯体系。加强重大动植物疫病防控，有效防控非洲猪瘟。深化南台农业合作，积极创建邵武省级农民创业园。持续做好武夷山农村一二三产业融合发展先导区、延平畜禽粪污资源化利用整县推进、光泽圣农白羽肉鸡原种场鸡育繁推一体化示范等项目申报工作，争创武夷山国家级现代农业产业园。强化乡村专业技术人才队伍建设，实施乡村本土人才能力提升工程，培育4000名新型职业农民。

全力建设美丽乡村。深化“五个留住”理念，统筹推进乡村规划设计和风貌营造，打造“一带N点”模式示范。加快建设宜居乡村，深化农村人居环境整治三年行动，推进“千村整治、百村示范”美丽乡村工程，再打造25个精品示范村；实施厕所革命，新建改造49座乡镇公厕、113座农村公厕；实施农村垃圾治理行动，深化“万人保洁”机制，开展“消灭垃圾”攻坚战，所有建制村建立生活垃圾治理常态化机制；实施农村污水治理行动，所有乡镇污水处理设施基本建成并投入使用；实施农房整治行动，每个县（市、区）选择1个乡镇作为试点，设立试点村，打造一批农房整治优质典型村庄；实施村容村貌提升行动，推进合福高铁南平段沿线村庄整治，加快村庄绿化美化，新改建“四好农村路”260公里。坚决清理整治“大棚房”，切实遏制农地非农化。加强农村精神文明建设，打造“一中心一所一服务站”，培育良好家风、文明乡风、淳朴民风。

深化农业农村改革。推进要素保障改革，巩固完善农村基本经营制度，深化农村土地“三权分置”，建立县级土地流转服务平台，加快土地确权和流转。进一步深化集体产权制度改革，形成权责明确的资源产权体系。加快转变乡镇职能，加强村级组织建设，推进乡村治理体系和治理能力现代化。

推动新时代“南平机制”创新发展。在更高层面、更宽领域探索赋予“南平机制”新内涵，将资金、资源、技术、人才等要素导入农村，激活乡村细胞，助推乡村振兴战略实施。推动下派村支书与科特派等多支下派队伍互动联动，深化“找好一条路子、抓好一个班子、建好一个机制、打好一个基础”的“四个一”工作机制。以连续推进科特派制度20周年为契机，加快成立闽北乡村振兴发展研究院，建设综合服务平台，支持科特派通过技术入股、成果转让、领办企业等方式与农民、企业结成利益共同体，推动科特派工作持续走在全省全国前列。

（七）保护优先、示范引领，加强生态文明建设

以更大力度、更实措施推进生态文明建设，全面提升生态环境质量。

坚决打好污染防治攻坚战。全力打赢蓝天保卫战，强化区域联防联控，加强能源、工业、建设、交通等领域治理，推进重点行业“散乱污”企业挥发性有机物排放综合整治。着力打好碧水保卫战，落实河湖长制，深化推进闽江流域、小流域和城市生活污水等综合治理，开展河流“清四乱”专项行动，全面完成县级集中式饮用水水源地环境问题整治。扎实推进净土保卫战，加强土壤污染管控和修复，完成农用地土壤污染详查，有序推进农用地土壤环境质量类别划定，建立污染地块清单和优先管控名录，加快推进垃圾分类处理和非正规垃圾堆放点整治，强化固体废物污染防治，打造清洁家园。

强化生态系统保护修复。落实主体功能区战略布局，全面完成全市生态保护红线、永久基本农田、城镇开发边界三条控制线划定工作，把该开发的地方高效集约开发好，把该保护的区域严格保护好。持续推进闽江流域山水林田湖草生态保护修复试点工程，加快实施富屯溪、建溪等流域生态保护修复项目。强化地质灾害工程治理，加大水土流失治理力度，综合治理水土流失面积25万亩以上；加强土地和矿产资源领域移动执法监察，严厉打击毁林种茶行为，建立健全长效管控体系。深入开展国土绿化行动，持续实施国家储备林质量精准提升工程，优化林分空间结构，完善森林生态功能，实现国家森林城市创建目标。

深化生态文明试验区建设。持续推进武夷山国家公园体制试点建设，深入实施自然资源产权制度改革，落实自然资源统一确权登记办法。加快完善环境监管体系，建立健全生态环境损害责任追究、污染物排放许可、环境信用评价、环境污染强制责任保险等制度。全面推行自然资源资产负债表编制、领导干部自然资源资产离任审计、生态系统价值核算等制度，继续开展生态文明建设目标评价考核工作，更加全面衡量绿色发展的质量和效益。

（八）勇立潮头、奋楫扬帆，深入推进改革开放

因循守旧没有出路，畏缩不前坐失良机。要继续推进一批重点领域和关键环节改革，推动更高水平的内外联动和双向开放。

强力推动改革。不断深化“互联网+放管服”改革，推动审批服务线上线下融合，将办事时限统一压缩至法定时限的50%以内，审批环节压缩至3个以内。加快推进国资国企改革，进一步完善现代企业制度，加强国有企业资产负债和负债率管控，积极发展混合所有制经济，加快国资监管职能转变，促进国有资本做强做优做大。深化投融资体制改革，促进政府投融资平台市场化转型。推进市县财政事权和支出责任划分改革，做到支出责任与财政事权相适应。坚决打好防范化解重大风险攻坚战，加强对政府债务风险和金融风险等重点领域风险防范和处置，坚决守住不发生系统性金融风险的底线。全面实施预算绩效管理，强化预算绩效管理监督问责和工作考核，着力提高财政资源配置效率和使用效益。统筹推进市县机构改革，加快建立健全适应政府机构改革职能转变之后的工作运行机制。

持续扩大开放。更加主动融入“一带一路”，实行更加精简、快捷的通关作业，加大国际和港澳地区航班航线培育力度，发挥海外侨社、友城以及香港南平联谊总会、澳门南平联谊会、武夷之友等平台作用，扩大对外开放“朋友圈”。深化南台交流，推动经济文化多层次合作。主动融入闽东北协同发展区，参与组建混合所有制的闽东北城市协作开发集团和“福建两翼发展投资基金”，以市场化方式积极推动闽东北区域交通、能源、信息等重大设施的统筹规划和协同建设，加强文化、体育、卫生与健康、职业教育等领域合作，推动建立闽东北区域旅游联盟，促进区域优势互补、共同繁荣。

（九）以人为本、关注民生，维护群众根本利益

牢固树立以人民为中心的发展思想，多办为民实事，多解民生难事，不断提升人民群众的获得感、幸福感、安全感。

强化社会保障。推进城乡统筹就业，城镇新增就业1.75万人，城镇失业人员再就业1.3万人。全面实施全民参保计划，完善机关事业单位人员、企业职工和城乡居民养老保险间的接续政策，推动建立更加公平、更可持续的养老保险制度。不断完善医疗保障制度，进一步提高医疗保障覆盖面。加强城乡低保、特困供养等社会救助，保障残疾人权益，关爱农村留守儿童。加强住房保障，支持房地产企业从销售商品房向“售租并举”转变。

完善公共服务。加大社会事业领域的补短板力度，不断增强发展的全面性、协调性和平衡性。优先发展教育，全市普惠性学前教育资源覆盖率扩大至80%，完善中小学教育质量评价体系，90%公办高中完成达标建设，高中阶段毛入学率达94%，加快对接全省新一轮初中毕业考试与高中招生制度改革，落实高考综合改革，持续推进高等教育内涵建设，支持武夷学院硕士学位授予单位培育建设。实施“健康南平2030”行动，坚持“三医联动”，统筹配置医疗卫生资源，建设县域“三大平台、六大中心”，引导优质医疗资源下沉，形成顺畅的双向转诊机制，解决群众看病难、看病贵问题。创新养老服务模式，推进医养结合，不断完善养老服务配套设施，建设一批居家社区养老服务照料中心和200个农村幸福院，推进乡镇敬老院社会化运营，新增养老床位1000张。推进新时代文明实践中心试点工作，加快县级融媒体中心和基层综合性文化服务中心建设，开展“百姓大舞台”文化惠民演出，进一步健全公共文化设施网络，丰富人民群众文化生活。全面启动第十七届省运会、第十一届老健会筹备工作，加快体育场馆建设，开展全民健身活动，继续办好武夷山国际马拉松赛、中国龙舟公开赛等赛事，积极创建全民运动健身模范市。完善公共法律服务体系，加大法律援助力度。认真做好第四次全国经济普查、第三次全国国土调查工作。

创新社会治理。践行社会主义核心价值观，深化群众性精神文明创建活动，提高群众文明素养和全社会文明程度。纵深推进扫黑除恶专项斗争，学习“枫桥经验”，深化“平安南平”系列行动，持续推进“四无”平安村（居）创建活动，扎实开展“七五”普法，完善县、乡、村三级矛盾纠纷多元调处平台。深入推进执法司法规范化建设，进一步开展案件评查和执法检查，提高执法司法水平。抓好综治信访维稳，加强社会矛盾排查化解，维护群众合法权益。完善公共信用信息平台，推进社会信用体系建设。落实安全生产责任，健全防灾减灾救灾体系，加大隐患排查治理力度，坚决遏制重特大事故发生。加强食品药品安全监管，进一步健全食品药品质量追溯制度，让老百姓吃得放心。

同时，做好国防动员、人民防空、国家安全等工作，落实双拥优抚安置政策，完善退役军人服务体系建设，推动军民融合深度发展。继续发展民族宗教、外事侨务、台港澳事务、档案方志、气象防震、老区库区等事业，支持工会、共青团、妇联等群团组织开展工作。

三、不忘初心、牢记使命，全面加强政府自身建设

全市各级政府要以习近平新时代中国特色社会主义思想为指引，认真落实省委“八个坚定不移”要求，以严实作风推进全面从严治党，深入转变职能，提升履职水平。

（一）坚持党的领导，提升政治站位。始终把党的政治建设摆在首位，持续推进“两学一做”学习教育常态化制度化，深入学习廖俊波精神，牢固树立“四个意识”，坚定“四个自信”，坚决维护习近平总书记党中央的核心、全党的核心地位，坚决维护党中央权威和集中统一领导，紧跟习近平总书记步伐，紧扣党中央节拍，严守政治纪律和政治规矩，坚决执行省委、市委的决定，确保各项工作沿着正确方向前进。

（二）坚持依法行政，提升法治水平。坚决维护宪法权威，保证宪法和法律实施，推进政府立法能力建设，加强地方性法规的实施，始终在法治轨道上开展工作。稳步推进政府法律顾问制度，建立科学的法治建设指标体系和考核体系。坚持科学、民主、依法决策，深化政务公开，依法接受同级人大及其常委会的监督，自觉接受政协的民主监督，主动接受社会和舆论监督，认真听取人大代表、政协委员意见，听取民主党派、工商联、无党派人士和各人民团体意见，让权力在阳光下运行。

（三）坚持开拓创新，提升行政能力。切实担负起落实赶超的政治责任，面对新形势新挑战，紧盯全年目标，发扬斗争精神，敢于亮剑、敢于担当，不在困难面前低头，不在挑战面前退缩；增强斗争本领，拓展对产业、金融、外贸、规划、建设等专业的学习，努力以知促行，以行促知。聚焦经济社会发展的重点难点问题，成立专项攻坚小组，专人负责、挂图作战，不见成效不收兵。把雷厉风行和久久为功有机结合起来，进一步解放思想、锐意进取，只要符合中央精神、本地实际和群众意愿的事项，就大胆试、放手干。继续总结提升“大督查”工作做法，有效推动工作落实。建立健全激励机制、问责机制和容错纠错机制，激发干部在新时代新担当新作为。

（四）坚持从严从实，提升政府形象。严格落实中央八项规定及其实施细则精神和省市实施办法，自觉传承和发扬 “马上就办、真抓实干”“四下基层”等优良作风，强化机关效能建设，以“踏石留印、抓铁有痕”的劲头，切实把功夫下到察实情、出实招、办实事、求实效上。巩固拓展中央巡视整改成果，树立正确政绩观，减少不合理的监督检查，力戒形式主义、官僚主义，把干部从无谓的事务中解脱出来。严格用制度管权管事管财管人，抓好工程招投标、政府采购、土地出让、资金使用等重点领域廉政风险防控，加大对重点项目、关键部门的审计监督。坚持无禁区、全覆盖、零容忍，严厉惩治群众身边的不正之风和腐败问题，建设廉洁政府。

各位代表！核心凝聚力量，思想指引方向。让我们更加紧密地团结在以习近平同志为核心的党中央周围，高举习近平新时代中国特色社会主义思想伟大旗帜，敢于担当，务实拼闯，为南平加快绿色发展推动高质量发展落实赶超而努力奋斗！
